# The Role of Group Dynamics in Scientific Inconsistencies: A Case Study of a Research Consortium

**DOI:** 10.1371/journal.pmed.1001143

**Published:** 2011-12-13

**Authors:** Judith G. M. Rosmalen, Albertine J. Oldehinkel

**Affiliations:** Interdisciplinary Center for Psychiatric Epidemiology and Graduate School of Medical Sciences, University of Groningen, University Medical Center Groningen, Groningen, The Netherlands

## Abstract

Judith Rosmalen and Albertine Oldehinkel describe their experience in the TRAILS research consortium to discuss why research teams might publish contradictory or inconsistent results despite procedures to avoid this; they emphasize the role of internal group dynamics rather than faulty publication practices.

**Summary Points**

- Findings reported in published articles often provide an unrealistic picture of the data that are actually generated in scientific research.
- This is partly due to ineffective group dynamics within research groups and collaborations.
- These practices may be improved by having clearly defined overall goals, explicitly described roles and responsibilities for all coauthors, and a rational choice of methodological strategies.

## Introduction

In October 2008, *PLoS Medicine* published a provocative paper by Young, Ioannidis, and Al-Ubaydi that discussed why current publication practices may distort science [1]. Based on economical insights and principles, Young and colleagues showed why and how the current system of publication provides an unrealistic picture of the data that are actually generated in scientific research. However, we believe that the problems they discussed arise not only at this macro level, but also at a lower aggregation level, that is, within research consortia. We feel it is time for scientists to also critically evaluate their own role, and acknowledge that group dynamics within research groups and collaborations might contribute to the persistence of problematic scientific practices.

This essay aims to highlight reasons why research groups might publish apparently contradictory or inconsistent results, by drawing upon our own experiences in a large longitudinal study. In particular, we wish to emphasize the potentially biasing effects of internal group dynamics, as opposed to the faulty publication practices that are more often discussed in the literature. We provide an analysis of group processes that can contribute to the publication of inconsistent findings in epidemiological cohort studies, and illustrate this analysis with observations made in our longitudinal survey TRacking Adolescents' Individual Lives Survey (TRAILS). TRAILS (e.g., [2]) is a well-designed and well-managed study in which several procedures were adopted to promote consistency, to prevent data-fishing expeditions, and to encourage the publication of null findings (see Box 1). Yet, in hindsight, we realize these procedures have not precluded publication of partly confusing and possibly irreproducible research findings, which have not significantly advanced our knowledge of the phenomenon under study. We hope that the recommendations based on our experiences in TRAILS, with which we will end this article, may serve various other research consortia as well.

**Box 1. TRAILS Procedures for Output Quality Enhancement**

- In order to get access to the consortium's data, publication plans with prespecified analyses must be submitted to and approved by the TRAILS management team.
- Only the data requested in the publication plans are provided to the authors.
- Lack of significant findings is not considered a valid argument to withdraw a publication plan.
- Outcome domains are directed by domain holders, who have rights to veto, check, and coauthor publications within their domain, and hence to protect coherence and consistency.
- Regular meetings are organized to exchange research plans and results.

### The Case: The Association between Salivary Cortisol and Mental Health in TRAILS

TRAILS is a multicenter study designed with the aim of finding the origins of mental health problems. Dysregulation of the hypothalamus-pituitary-adrenal axis was an interesting candidate, and several TRAILS papers were published on the relationship between cortisol and various aspects of psychopathology. However, it appeared that it was not possible for TRAILS to make a comprehensible synthesis regarding the potential role of cortisol in the etiology of psychopathology. Concerned by this observation, we analyzed the strategies used by the consortium to answer the questions on cortisol and psychopathology, and found that, although the strategies employed within the papers were usually correct, there were inconsistencies across papers. These inconsistencies concerned the operationalization of psychopathology (different questionnaires, informants, cutoff levels), the cortisol variables (different composite measures), and the use of statistical methods and included confounders. The end result was a rather confusing pattern of findings. For instance, self-reported oppositional defiant problems were cross-sectionally associated with high morning cortisol levels in girls, and parent-reported disruptive (i.e., oppositional defiant plus conduct) problems predicted high evening cortisol levels in boys (for details see Table 1). In general, the results could not be combined in an overarching model, and were thus disappointing with regard to scientific progress. In contrast, the end result in terms of publication output was quite positive: the majority of papers were presented at international conferences and published in highly cited journals (Table 1), and several students earned PhD degrees based on their work on the subject.

**Table 1:** Publications on associations between (basal) cortisol measures and psychopathology in TRAILS.

Domain	Study	Mental Health Measures	Cortisol Measures	Covariates	Tests^a^	Significant Associations
**Anxiety**	[9]	T1 RCADS anxiety score (root-transformed); categories: never, only preschool, only current, persistent anxiety	Cort_0700_; Cort_0730_; Cort_2000_; AUC_g_ (root-transformed)	Gender, age, pubertal stage, T1 depression (root-transformed)	20	1. Persistent vs. current anxiety: Cort_0700_ ↑; 2. Persistent vs. no/current anxiety: AUC_g_ ↑
	[10]	T2 RCADS anxiety score; categories: persistently low, increaser, decreaser, persistently high	AUC; Cort_2000_ (root-transformed)	Gender, T2 depression	8	1. Persistent high vs. rest: Cort_2000_ ↓; 2. Increasing anxiety vs. rest: AUC ↑
	[11]	T1 RCADS anxiety score; T2 RCADS anxiety score	AUC_g_ (root-transformed)	T1 anxiety, T1 and T2 depression, pubertal stage, autonomic measures	30	1. Girls, parental INT: AUC_g_—T2 Anx ↑
**Depression**	[12]	T1 YSR affective problems: total score, somatic symptoms and cognitive-affective symptoms	AUC_i_	Gender, age, physical activity, physical health, BMI	5	1. Boys: YSR somatic—AUC_i_ ↑; 2. Boys: YSR affective—AUC_i_ ↓;
**Disruptive behaviors**	[13]	T1 CBCL ADH, OD, CD scores; T1 YSR ADH, OD, CD scores; T1 ASBQ score	Cort_0700_; Cort_0730_; Cort_2000_; AUC_g_	Gender, age, pubertal stage, BMI	84	1. Total group: YSR ADH—Cort_2000_ ↑; 2. Girls: YSR ADH—Cort_2000_ ↑; 3. Girls: YSR OD—Cort_0730_ ↑; 4. Boys vs. girls: CBCL ADH—Cort_0730_ ↑; 5. Boys vs. girls: YSR OD—AUC_g_ ↓; 6. Boys vs. girls: ASBQ—AUC_g_ ↓
	[14]	T2 CBCL DB score; T2 YSR DB score	AUC_g_; Cort_2000_;	Gender, pubertal stage, SES, T1 DB, T1 INT	36	1. Boys: Cort_2000_—T2 CBCL DB ↑; 2. High T1 DB: AUC_g_—T2 YSR DB ↓
	[15]	T1 mean CBCL/YSR EXT, INT scores; categories: control, pure EXT, pure INT, both; severity and directionality scores	AUC_g_; AUC_i_; Cort_2000_	Gender, sampling month	18	1. Total group: pure EXT—AUC_i_ ↑; 2. Total group: EXT vs. INT—Cort_2000_↑; 3. Girls: pure EXT—AUC_i_ ↑
**Psychosis proneness**	[16]	T3 CAPE positive symptoms score	AUC_g_, AUC_i_, Cort_2000_	Gender, pubertal stage	9	None
**Substance use**	[17]	Cannabis use categories: non-use, early-onset, late-onset	Cort_0700_; Cort_0730_; Cort_2000_	Gender, pubertal stage	9	1. Early vs. late users: Cort_0730_ ↓; 2. Early vs. non-users: Cort_2000_↑; 3. Late vs. non-users: Cort_2000_↑
	[18]	T1 ever smoking; T1 ever drinking; T2 ever smoking; T2 ever drinking; T2 number of cigarettes per week; T2 number of drinks per week	Cort_0700_; Cort_0730_; Cort_2000_; AUC_i_	Gender, age, maternal risk, T1 substance use	24	1. Cort_0700_—T1 ever smoking ↓; 2. Cort_2000_—T1 ever smoking ↑; 3. AUC_g_—T1 ever smoking ↑; 4. Cort_0700_—T2 number of cigarettes ↑; 5. Cort_0730_—T2 number of cigarettes ↑

a Number of tests reported in the article, in the total sample as well as in subgroups. Including preliminary tests, if reported.

ADH, attention deficit hyperactivity; ASBQ, Antisocial Behavior Questionnaire; AUC_g_, area under the curve with respect to the ground; AUC_i_, area under the curve with respect to the increase; BMI, body mass index; CAPE, Community Assessment of Psychic Experiences; CBCL, Child Behavior CheckList; CD, conduct disorder; Cort_0700_, cortisol directly after waking up; Cort_0730_, cortisol half an hour after waking up; Cort_2000_, cortisol at 8 pm; DB, disruptive behavior (oppositional defiant plus conduct disorder); EXT, externalizing problems; INT, internalizing problems; MDD, major depressive disorder; OD, oppositional defiant; RCADS, Revised Child Anxiety and Depression Scale; YSR, Youth Self-Report.

We were interested in the processes that enabled the publication of incoherent papers. To identify these processes, we made an overview of all relevant studies on cortisol and psychopathology and discussed this at a meeting of TRAILS researchers, which yielded several potential causes of the problem. The identified causes were analyzed, and the resulting report was sent to all authors of the cortisol articles listed in Table 1, with the request to indicate which processes they experienced, which processes they did not experience, and whether the report missed any processes they thought were important. The authors' comments were incorporated into our analysis reported here.

**Figure 1:**
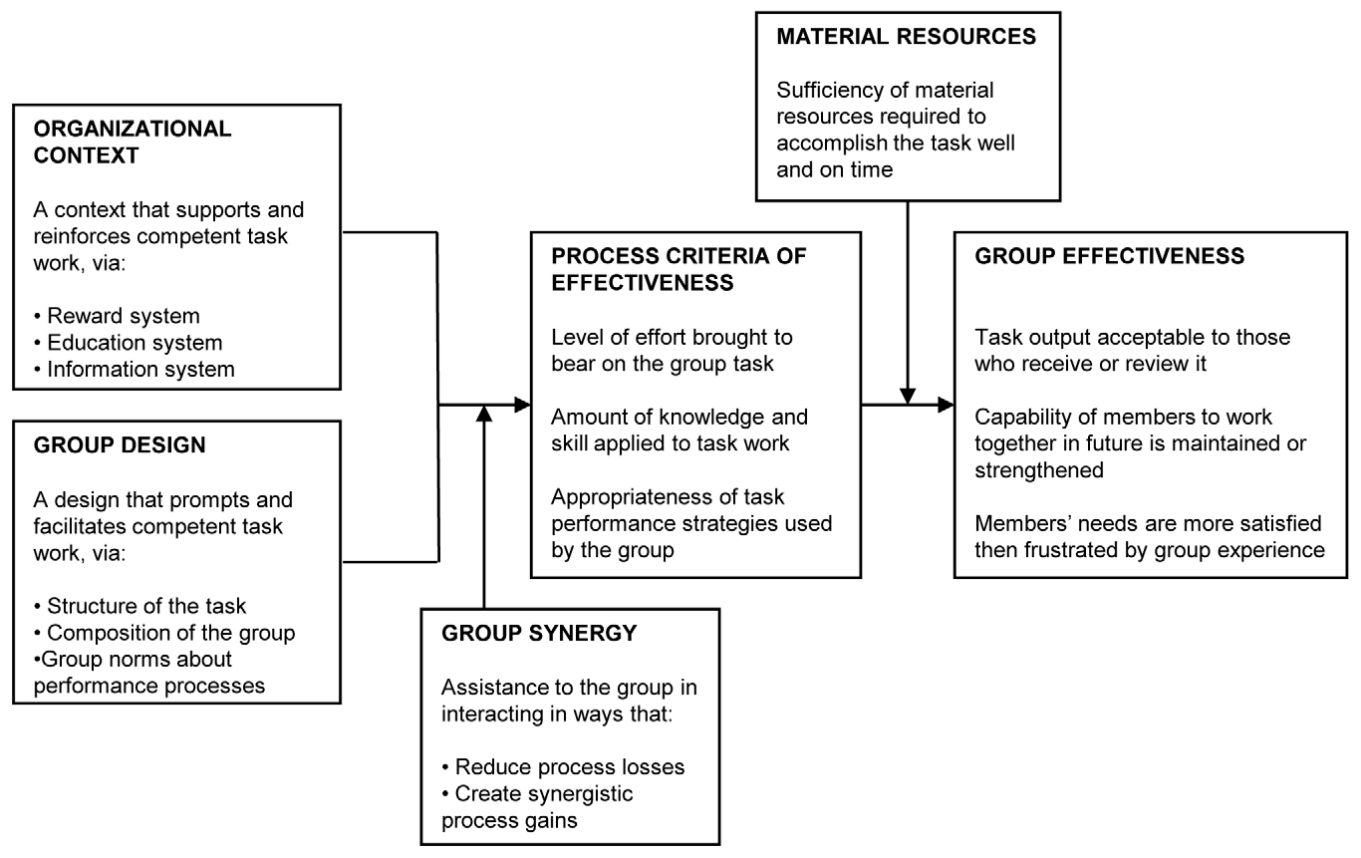
Hackman's normative model of group effectiveness.

The identified causes were analyzed using Hackman's normative model of group effectiveness [3],[4] as a general framework. This model is a theoretical framework incorporating social psychological knowledge derived from descriptive research on group performance, with the aim to guide improvements. Although we realize that there are other ways of looking at social interactions that might also apply here, two characteristics of Hackman's model make it particularly interesting for our case study. First, Hackman uses a multidimensional definition of group effectiveness, which includes not only task but also group outcomes. A highly effective group in this model is thus characterized by a tight balance between an appropriate amount of work-related criticism and good social relations. Second, Hackman suggested that the model can provide a basis for diagnosing the strengths and weaknesses of groups, and can thus be used as action guide in the world of practice [3]. Hackman hypothesized that group effectiveness is determined by an interaction between organizational context and group characteristics (Figure 1). While Young et al.'s paper discussed the organizational context, we will analyze these group characteristics that may influence effectiveness, in particular the design and synergy of the group.

### Group Design

Three important aspects of group design can be distinguished in Hackman's model: (1) the structure of the task, (2) the composition of the group, and (3) group norms about performance processes.

#### The structure of the task

In our case, the task involved elucidating the relationship between cortisol and psychopathology. The structure of the task was sliced over different projects, often PhD projects, each approaching a sub-question regarding a specific domain of psychopathology. In order to protect the coherence of and prevent overlap between simultaneous PhD projects, PhD students had to draft a plan for their whole dissertation (usually four to six articles in the Netherlands), including plans for further analysis of the primary relations that were expected to be found in the data.

What was meant to protect PhD students became a pitfall when the initial cross-sectional analyses yielded mostly null or very weak findings. Pushed by the pressure to produce publishable papers, and by the inconsistencies between the negative findings in TRAILS and the significant associations reported in the literature, various measures of the predictor and the outcome variables were explored, varying sets of potential confounders were included, and the data were analyzed using divergent statistical techniques. Variation in measures and methods used was further increased by reviewers' preferences, which, for obvious reasons, were met as much as possible. Thus, the task as a whole was not always approached in a coherent and logical manner.

#### The composition of the group

Each sub-question had its own team of authors, often partly overlapping with other teams. Most publications involved coauthors from various research institutes, with sometimes long-standing collaborations. The desire and need to maintain good relations with colleagues is likely to affect the role of coauthors at times: critical evaluation of each others' ideas and work is not always appreciated, particularly not when it delays or even endangers the production of a paper. Rather than discussing divergent opinions until consensus is reached, the final decisions regarding a manuscript are usually made by the researchers with the strongest interests to get the paper published: the first, second, and last author. Some of the resulting methodological inconsistencies might be prevented by an overall scientific director with decisive power. However, a hierarchical structure like that tends to be at odds with academic freedom and autonomy, which are highly cherished values in science, and differences of opinion on the best research strategies are the rule rather than the exception.

#### Group norms

The TRAILS consortium consists of investigators who maintain high quality standards for themselves and their research groups. Also with regard to the cortisol analyses, every member of the consortium agreed that, in principle, high-quality research as well as consistency and replicability of the findings were important goals. But these goals are not as self-evident as one might hope, and there is a grey area between “indisputably excellent” and “clearly not up to the mark.” Hence, in practice, the pressure to produce output may be hard to reconcile with the above-described goals and tends to make at least a subset of the authors willing to allow some compromises in order not to delay the submission of an article. Together with the before-mentioned desire to maintain good interpersonal relationships within a consortium, this suggests that group members are sometimes not very likely to obstruct or criticize each other with regard to methodological choices or inconsistencies.

### Group Synergy

Group synergy arises if group processes result in gains in energy and effectiveness that go beyond what would be expected given the organizational context and the task design. In groups with high synergy, members think of themselves as group members first and as individuals second. In groups that produce scientific papers within the context of large longitudinal cohort studies, group synergy is not easy to obtain. Most PhD students will leave the group after they obtain their PhD, so their primary goal is individual: obtaining a PhD. Senior investigators also tend to have individual goals, because in the end their own performance is being evaluated, not the group performance, and they all have their own responsibilities towards their faculties and grant funders. In short, the strong focus on individual achievements in science hampers group synergy, particularly in multicenter collaborations. On the other hand, for many of the current scientific questions, research consortia are necessary because of the amount of money, participants, or expertise needed. The fundamental problem is the way science manages cooperation versus competition.

## Recommendations

### The Task

Although current scientific practices sometimes seem to suggest otherwise, the overarching task of a research group is not to write papers, but to address scientifically relevant issues. This often involves multiple papers. To avoid that the writing of a particular paper starts to take on a life of its own, it is important that the overarching task also be considered a meaningful piece of work, and that its organization be appointed to a steering committee with clearly defined goals, rights, and responsibilities, which are agreed upon by all participating investigators beforehand. Explicit, and preferably written, general agreement on the overall strategy will increase the likelihood that individual writing teams act in accordance with the overarching goals. In addition, regular general research meetings can be organized to discuss specific papers in relation to the overall strategy. This strategy will increase not only the conceptual and methodological consistency within consortia, but also the shared commitment and possibilities to learn from each other.

### The Writing Teams

For individual articles, the appropriate number of authors is, in general, not too many. Coauthors are the ones who design, analyze, and write. Other, more general contributions to studies such as grants, project management, delivering research subjects or materials, and laboratory work, which are often rewarded with honorary authorships, should be valued as important scientific achievements in another way, in order to take away the impetus to grant coauthorship to contributors who do not actually participate in the writing process. Writing a scientific article together requires a high level of task interdependence among team members. If individuals' work becomes intertwined with that of others, however, it is more difficult for them to determine a sense of personal achievement, and they will be unlikely to make extraordinary efforts unless they view their individual task as meaningful [5]. This has clear implications for the organization of teams of coauthors: it is important to start the writing process by clarifying the unique role, responsibility, and contribution of each coauthor, and how this role will be filled. An advantage of explicitly recognizing members' expertise and role within the group is that group members are thus mutually aware of each other's areas of expertise from the beginning onwards, which has been shown to increase use of members' expertise and information exchange [6].

Optimal teams consist of coauthors whose skills are, to a large extent, complementary rather than overlapping. Whereas heterogeneity of coauthors, providing it is within reasonable bounds, is likely to improve performance, too much overlap in skills will promote social loafing, i.e., the tendency to reduce individual effort when working in teams compared to when working alone [5],[7]. Non-overlapping skills will also increase the task visibility of the coauthors, i.e., individuals' belief that their efforts are seen as important by others [5]. All ideas should be evaluated thoroughly, regardless of the status and seniority of the author. Finally, team members should be encouraged to teach and learn from each other, and so increase the quality of the writing team, as well as of the research group as a whole: development of knowledge, skills, and talent is a scientific obligation.

### The Methodological Strategy

Researchers who participate in large longitudinal studies should be aware of the necessity of building explicitly on prior work performed on the same dataset. In practice, this implies using similar methods, unless there are strong theoretical reasons to do otherwise (statistical significance is not among these reasons), which should be clearly outlined in the papers involved. Importantly, this means that the overall strategy should be agreed upon before the first paper is published. Post hoc or irrational choices about the inclusion of covariates can be prevented by choosing the covariates to be included before starting the analyses, e.g., based on causal graphs, which provide an excellent guideline for the selection of potential confounders (e.g., [8]).

We acknowledge that science is intrinsically unpredictable. It will be impossible to account for all possibilities in advance, since part of the scientific process involves coming up with new ways to measure phenomena. Nevertheless, even if no single best strategy can be specified in advance, it is possible to build group norms that increase the likelihood that members will develop task-appropriate performance strategies and execute them well. For instance, consortia could agree upon a number of standard methodological papers and evaluate every publication proposal and paper against the recommended methodological guidelines. It is essential that every consortium member explicitly agree with these standard papers, in order to ensure that the performance strategies become part of the fabric of the group. Once a strategy is agreed upon, group members tend to behave in accordance with it and enforce adherence to it. Thus, establishing group norms is important because they can foster self-regulation and reflection, and thereby bring science to a higher level.

A more rigid way to avoid methodological divergence could be to have all analyses performed by an independent statistician. Apart from the fact that this is not always financially feasible, an evident downside of this system is that PhD students would not be trained to do their own analyses, which we feel is an important aspect of their education as independent researchers. We do recommend, however, discussing the analytic plan and having all papers reviewed before submission by an independent statistician. Additionally, for reviewers evaluating a paper, it would be helpful if cohort studies generating multiple papers had a unique code in journal literature search systems (e.g., PubMed), so that it would be easy to find all papers published previously on a certain cohort to check for methodological consistency.

## Conclusion

For most researchers, the ultimate goal of science is to approach the best description of the “truth,” but scientific quality is evaluated in terms of publications or citations. The best description of the truth does not always have the best odds of being published in a high-ranking journal or being frequently cited. One solution would be to change the evaluating system, and we strongly agree with the suggestions made by Young et al. in this regard. However, macro-level processes are hard to change because that requires action from anonymous others outside our sphere of influence. Micro-level processes are more malleable, and changes at this level can be implemented right away. That does not mean that micro-level processes are easy to change. Perhaps the most important counterforce is an unbalance between those who benefit and those who pay the costs of misbehavior with regard to publication practices. The benefits (high scientific output) accrue to individual researchers or research teams—exactly the ones who should change their behavior—while the costs (low scientific progress) are borne by the entire world. Yet, continuing the status quo is unworthy of science in our view. We have provided a number of suggestions to increase consistency within research consortia. We would like to acknowledge that these recommendations are based on our experiences, and thus are grounded in practice rather than in theory.

This essay aims to highlight reasons why research groups might publish contradictory or incoherent results. To the best of our knowledge, this is the first analysis of this phenomenon that is illustrated with actual experiences in a research consortium. Scientific colleagues from various fields have read and commented on our analyses. Their overall opinion was that the processes described are common and recognizable, but it should be emphasized that this case study is based on a subjective and possibly arbitrary analysis, and that alternative interpretations are possible. Nevertheless, we hope our analysis will stimulate a broader discussion of problematic scientific practices, which include not only faulty publication practices but also the potentially biasing effects of internal group dynamics. In the end, both the system and the consortia are our own products and thus our shared but also individual responsibility.

## Abbreviations

TRAILS: TRacking Adolescents' Individual Lives Survey
